# Cost-effectiveness analysis of cetuximab for the treatment of head and neck cancers in India

**DOI:** 10.3389/fphar.2026.1948615

**Published:** 2026-10-23

**Authors:** Nidhi Gupta, Jyoti Dixit, Gaurav Jyani, Shankar Prinja

**Affiliations:** 1 Department of Radiation Oncology, Government Medical College and Hospital, Chandigarh, India; 2 Department of Community Medicine and School of Public Health, Post Graduate Institute of Medical Education and Research (PGIMER), Chandigarh, India

**Keywords:** cetuximab, cost-effectiveness analysis, incremental cost-effectiveness ratio, locally advanced head and neck cancer, quality-adjusted life years, recurrent and/or metastatic head and neck cancer

## Abstract

**Background:**

Head and neck cancers (HNC) comprise 5% of all malignancies worldwide. Cetuximab is the first targeted therapy to demonstrate a significant survival benefit in patients with locally advanced HNC (LAHNC) and recurrent or metastatic HNC (R/MHNC). In view of this, we undertook this economic evaluation to ascertain the costs and health benefits associated with (i) the use of RT + CTX over RT alone for LAHNC patients who are ineligible for cisplatin-based chemoradiotherapy and (ii) CT + CTX over CT alone for R/MHNC patients from the perspective of Indian healthcare system.

**Methods:**

A Markov model was constructed to assess the lifetime costs and health outcomes using cetuximab in combination with radiotherapy (RT + CTX) for treatment of cisplatin-ineligible LAHNC (*Analysis I*), and cetuximab in combination with platinum-based chemotherapy (CT + CTX) for treatment of R/MHNC patients in India (*Analysis II*). Using an abridged societal perspective lifetime costs and quality adjusted life years (QALYs) were evaluated, discounting future costs and consequences at 3% annually. For both the analyses, costs of original as well as biosimilar cetuximab were evaluated. The cost-effectiveness was assessed in terms of incremental cost effectiveness ratio (ICER) which was compared against a threshold of 1-time *per capita* gross domestic product (GDP) for India in 2024–25 (₹ 247,045; US$ 2,697).

**Results:**

Adding biosimilar cetuximab to radiotherapy for cisplatin-ineligible LAHNC patients resulted in 2.73 (95% CI: 2.11–3.30) QALYs, while adding biosimilar cetuximab to platinum-based chemotherapy for R/MHNC resulted in 0.78 (95% CI: 0.61–0.96) QALYs over the modelled lifetime horizon. The incremental cost per QALY gained was found to be ₹ 501,320 with use of RT + CTX and ₹ 15,023,037 with use of CT + CTX. At the currently recommended willingness to pay (WTP) threshold, treatment with cetuximab is not a cost-effective treatment option for patients with LAHNC and R/MHNC patients respectively in India. A 50% reduction in the price of biosimilar would make it a cost-effective option for treatment of LAHNC in India.

**Conclusion:**

Strategic purchasing of biosimilar cetuximab at reduced price can provide good value for money for treatment of patients with LAHNC.

## Key points for decision makers

- We found that addition of biosimilar cetuximab to radiotherapy for cisplatin-ineligible locally advanced head and neck cancer patients (LAHNC) resulted in higher quality-adjusted life years (QALYs) as compared to radiotherapy alone. However, this was associated with significant incremental costs.
- Addition of biosimilar cetuximab to platinum-based chemotherapy for recurrent/metastatic head and neck cancer (R/M HNC) patients also resulted in improved QALYs compared to chemotherapy alone, but at substantially high additional costs.
- None of the treatment strategies involving cetuximab were found to be cost-effective for the treatment of LAHNC and R/M HNC at the current price levels in India. A 50% reduction in the price of biosimilar cetuximab could make radiotherapy plus cetuximab a cost-effective treatment option for locally advanced head and neck cancer patients in India.
- Our study provides important evidence for price negotiation as part of broader strategic purchasing arrangements if cetuximab is to be included in the health benefits package under India’s flagship publicly financed health insurance scheme, *Ayushman Bharat Pradhan Mantri Jan Arogya Yojana* (AB PM-JAY).

## Introduction

Head and neck cancers (HNCs) are ranked seventh most common cancer worldwide and the third most common cancer in India (Lakshmi et al., 2025). Overall, 1.56 million new HNC cases and 874,404 deaths were reported in India in 2024 (Mathur et al., 2025). Nearly 90% of HNCs are squamous cell carcinomas which overexpress epidermal growth factor receptor (EGFR). EGFR overexpression is associated with aggressive behaviour, poorer outcomes and resistance to radiotherapy (RT).

More than 65% of HNCs in India present with locally advanced disease (LAHNC) (Bagal et al., 2023). Majority of these are unresectable and receive definitive chemoradiation (CTRT) with cisplatin which is the current standard of care (National Comprehensive Cancer Network, 2024; Health Benefit Package, 2022). However, approximately 30%–35% patients are ineligible for cisplatin-based chemotherapy (Sun et al., 2022). For such LAHNC patients who are ineligible for cisplatin based CTRT, addition of cetuximab with radiotherapy (RT + CTX) is recommended over RT alone (National Comprehensive Cancer Network, 2024). Cetuximab (CTX) is a monoclonal antibody against the ligand binding domain of EGFR, and hence improves the efficacy of treatment. Bonner et al. (Bonner et al., 2006; Bonner et al., 2010) demonstrated significantly prolonged median locoregional control (24.4 vs. 14.9 months), progression-free survival (17.1 vs. 12.4 months) and overall survival (49.0 vs. 29.3 months) for patients treated with RT + CTX compared to RT alone respectively (Bonner et al., 2006; Bonner et al., 2010).

Despite the aggressive management of LAHNCs, nearly 50% patients experience treatment failure as loco-regional recurrences (LRR) and distant metastatic (DM) disease or may present with primary metastatic disease with a median survival of less than 1 year (Rangaraju et al., 2012). Management of such recurrent and/or metastatic HNCs (R/MHNCs) that are inoperable or not amenable to salvage surgery or radiation involves systemic treatment with chemotherapy alone, targeted therapy with cetuximab with or without chemotherapy, or with immunotherapy such as pembrolizumab with or without chemotherapy (National Comprehensive Cancer Network, 2024; Parikh et al., 2025). In the EXTREME trial for patients with R/MHNCs, addition of cetuximab to platinum-based chemotherapy was associated with 2.3 months increase in progression-free survival (PFS) (P = 0.001), and a 2.7-month increase in overall survival (OS) (P = 0.036), compared to platinum-based chemotherapy alone (Vermorken et al., 2008; Vermorken et al., 2014).

Despite the evidence for improved clinical effectiveness of cetuximab, its access is poor in India in view of the high cost (Patil et al., 2021). One of the strategies to expand the access to treatment is to include it in the health benefits package of India’s government funded health insurance program–*Ayushman Bharat Pradhan Mantri Jan Arogya Yojana* (PM-JAY) (Prinja et al., 2020). As part of its decision-making, the evidence on its cost-effectiveness was sought from India’s Health Technology Assessment agency (Prinja et al., 2023a). Previous cost-effectiveness analysis for RT + CTX found it to be cost effective over RT alone for LAHNCs patients in five European countries (Brown et al., 2008), Netherlands (van et al., 2015) and Taiwan (Chan et al., 2011). On the other hand, cetuximab combined with platinum-based chemotherapy (CT + CTX) over chemotherapy alone (CT) for R/MHNC patients was not reported to be cost-effective in China (Lang and Dong, 2020), Canada (Hannouf et al., 2012) and United Kingdom (Greenhalgh et al., 2009). Considering the heterogeneity in evidence, differences in population characteristics, cost structures, and different willingness to pay (WTP) thresholds, generalizability of existing evidence to India is limited. Another important reason for undertaking India-specific analysis is the recent introduction of a biosimilar cetuximab.

In view of this, we undertook this economic evaluation to ascertain the costs and health benefits associated with (i) the use of RT + CTX over RT alone for LAHNC patients who are ineligible for cisplatin-based chemoradiotherapy and (ii) CT + CTX over CT alone for R/MHNC patients from the perspective of Indian healthcare system.

## Materials and methods

### Model Structure

A Markov model was constructed in Microsoft Excel to assess the lifetime costs and health outcomes. As shown in Figure 1, the model structure for *Analysis I* of LAHNCs comprised of five mutually exclusive health states, namely, progression free survival 1 (PFS1), loco-regional recurrence (LRR), distant metastasis (DM1), all-cause mortality (ACM), and disease-specific mortality (DSM1). The model structure for *Analysis II* of R/MHNC comprised of four mutually exclusive health states, namely, progression free survival 2 (PFS2), progressive disease (PD), ACM, and disease-specific mortality 2 (DSM2) as shown in Figure 2. Patients in PFS1 and PFS2 health states were assumed to die only due to natural causes and there are no cancer-related deaths. We assumed cancer-related deaths only from LRR, DM1, and PD health states.

**FIGURE 1:**
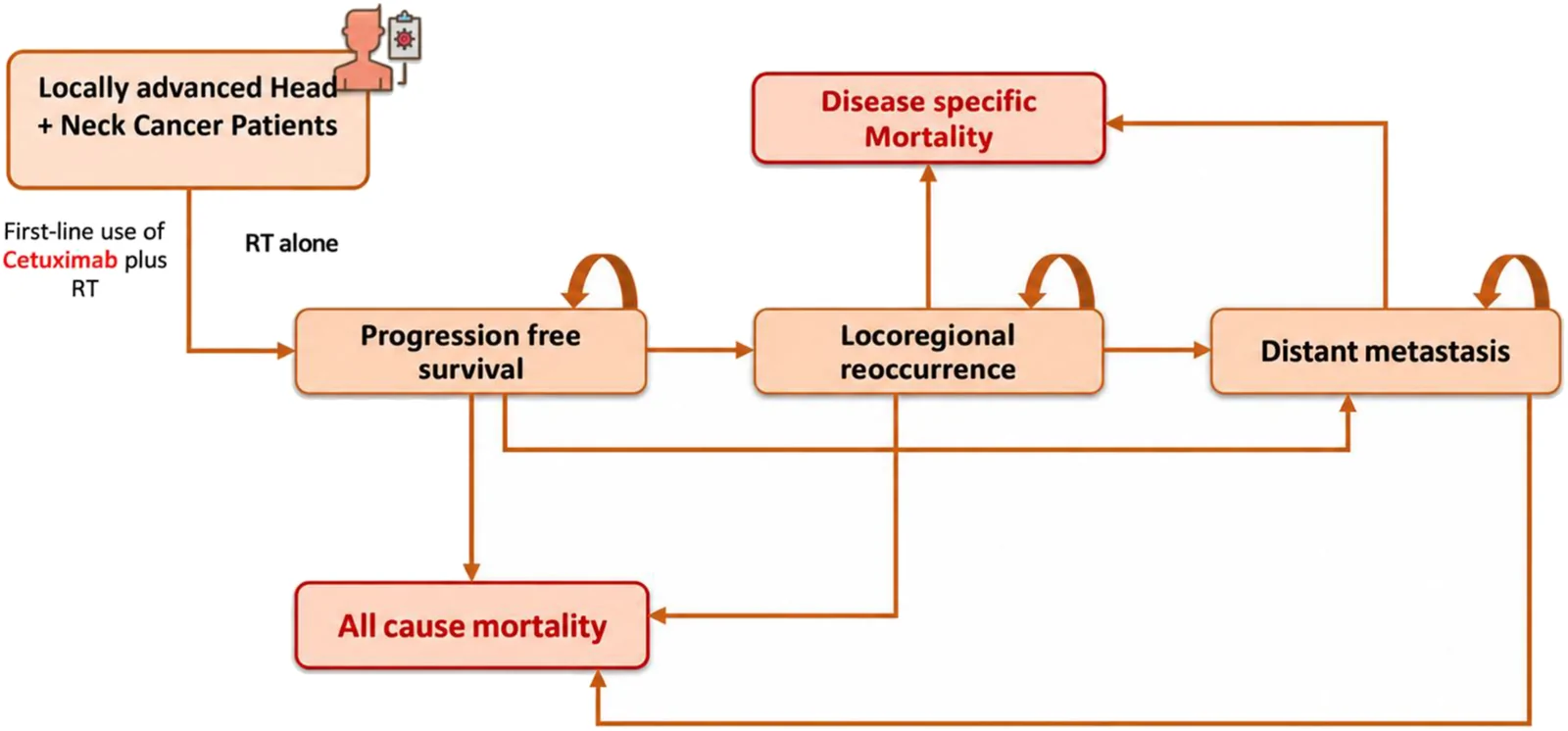
Model structure for LAHNC (analysis I).

**FIGURE 2:**
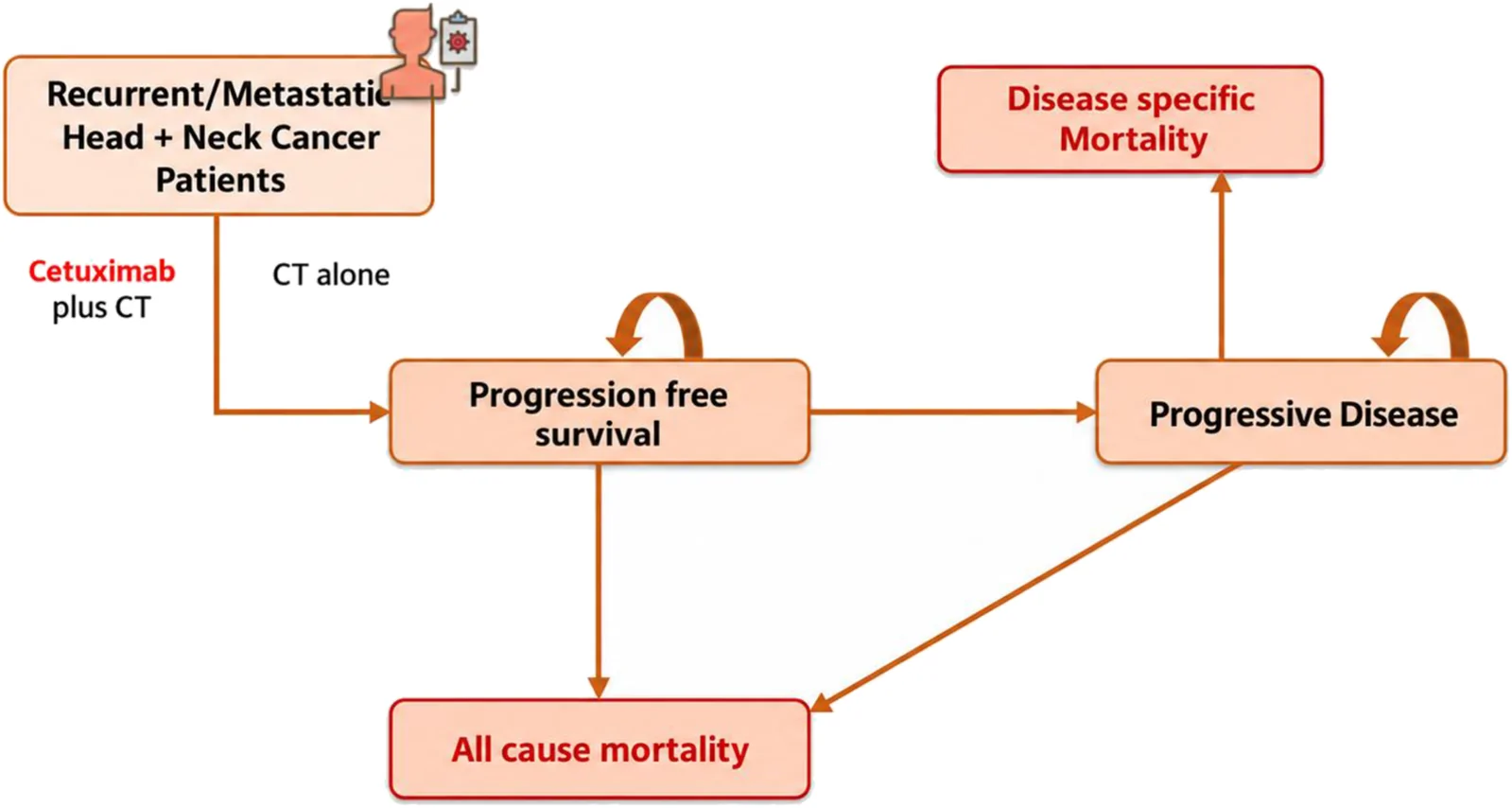
Model structure for R/MHNC (analysis II).

Both the models start with patients at 56 years of age, the median age of diagnosis for HNCs in India (Badola et al., 2023). The Markov cycle length was considered as 1 month for both the models, which is consistent with the Kaplein Meir (KM) curves provided in the published literature as well as with standard treatment protocol (National Comprehensive Cancer Network, 2024; Bonner et al., 2006; Vermorken et al., 2008). An abridged societal perspective was used as per standard Indian health technology assessment guidelines (HTAIn, 2021). Future costs and consequences were discounted at 3% annually for future time preferences of cost and utility (HTAIn, 2021). We did not include the indirect cost due to productivity losses, in line with the Indian reference case methodological guidance (HTAIn, 2021). We have followed the Consolidated Health Economic Evaluation Reporting Standards (CHEERS) to report the findings (Huse et al., 2013).

### Treatment sequences

*Analysis I*: Two treatment arms were modelled. (i) RT alone delivered as conventional fractionation once per day (70Gy/35 fractions/7 weeks) (ii) RT (70Gy/35 fractions/7 weeks) + CTX at a loading dose of 400 mg/m^2^ of body surface area (BSA), delivered 1 week before the start of RT, followed by 250 mg/m^2^ weekly for the duration of RT (Bonner et al., 2006).

*Analysis II*: Two treatment arms were modelled. (i) Cisplatin at a dose of 100 mg/m^2^ on day 1 plus fluorouracil at a dose of 1,000 mg/m^2^ per day for 4 days every 3 weeks for a maximum of 6 cycles (Vermorken et al., 2008); (ii) cisplatin at a dose of 100 mg/m^2^ on day 1 plus fluorouracil at a dose of 1,000 mg/m^2^ per day for 4 days every 3 weeks for a maximum of 6 cycles plus cetuximab at an initial loading dose of 400 mg/m^2^, followed by 250 mg/m^2^ per week till disease progression (Vermorken et al., 2008). We assumed a BSA of 1.6 m^2^ for calculating dosage of all drugs including cetuximab, cisplatin, fluorouracil. This was computed based on the weighted average height (170.2 cm) and weight (60.2 kg) of an average Indian adult, considering the gender distribution of the Indian population (National Nutrition Monitoring Bureau, 2017) and Indian reference anthropometric data (Ministry of Statistics and Programme Implementation and Government of India, 2023).

In *Analysis I*, we assumed that all patients entering the DM health state would receive systemic therapy in addition to palliative RT and supportive care, while systemic therapy would be administered to 70% of patients entering the LRR health state. The first line systemic therapy was assumed to be given to those who had progressed for the first time, i.e., patients who moved from PFS1 to LRR and PFS1 to DM1. Different systemic regimens (platinum-based chemotherapy, taxanes, immune check point inhibitors or targeted therapy given alone or in combination with chemotherapy, oral or injectable metronomic therapy) were administered as part of first line therapy. The first-line systemic therapy included paclitaxel (175 mg/m^2^) plus carboplatin (AUC 5) or cisplatin (100 mg/m^2^) administered every 3 weeks (25%), paclitaxel (175 mg/m^2^) administered every 3 weeks (20%), and cisplatin (100 mg/m^2^) D1 and Inj. 5FU 1000mg/m2 X D1-D5, administered every 3 weeks (25%). Other systemic treatment options (5%) included pembrolizumab (200 mg intravenously every 3 weeks until disease progression), nivolumab (3 mg/kg every 2 weeks until PD), pembrolizumab (200 mg) plus carboplatin (AUC 5) and 5-fluorouracil (5FU; 1,000 mg/m^2^ for 4 days) for 6 cycles followed by pembrolizumab 200 mg intravenously every 3 weeks until PD, and cetuximab (400 mg/m^2^ on day 1 followed by 250 mg/m^2^ on days 2 and 3) plus carboplatin (AUC 5 on day 1) and 5FU (1,000 mg/m^2^ on days 1–4) every 3 weeks for 6 cycles, followed by cetuximab until PD (1.25%). Oral metronomic therapy with methotrexate and celecoxib administered weekly was assumed for 15% of patients, while best supportive care was considered for 10% of patients.

After first line systemic therapy, all patients in PD health state were administered second line systemic therapy or palliative RT. Second-line treatment comprised best supportive care (50%), oral metronomic therapy with methotrexate and celecoxib administered weekly (20%), or single agent chemotherapy with carboplatin (AUC 5), paclitaxel (175 mg/m^2^), or docetaxel (75 mg/m^2^) administered every 3 weeks (30%).

The proportions for diagnostic investigations for diagnosis and response assessment, subsequent treatment, and resource utilisation were obtained through consultation with four clinical oncologists experienced in the management of HNC, working at high volume public hospitals, as patient-level data were unavailable (Supplementary Tables S3 and S4). These inputs were used to reflect expected treatment and resource-use patterns in routine clinical practice in the Indian context.

In *Analysis II*, patients with PD discontinued first-line systemic therapy and were assumed to receive second-line systemic therapy. Patients in PFS2 continued to receive first-line systemic therapy. After completion of 6 cycles of first-line systemic therapy, patients were assessed for response; those remaining in the PFS2 state continued with maintenance cetuximab (CTX) until PD, while patients with PD following chemotherapy or CTX maintenance were assumed to receive second-line systemic therapy PD following chemotherapy or CTX maintenance were assumed to receive second-line therapy comprising chemotherapy with paclitaxel (175 mg/m^2^) or docetaxel (75 mg/m^2^) administered every 3 weeks (30%), oral metronomic therapy (20%), or best supportive care (50%), in addition to palliative RT and supportive care.

### Valuation of consequences

We used data from the Bonner trial (Bonner et al., 2006; Bonner et al., 2010) for *Analysis I* and the EXTREME trial (Vermorken et al., 2008; Vermorken et al., 2014) for *Analysis II*. For *Analysis I*, the Bonner trial was selected because it provided phase III randomized evidence directly comparing RT + CTX with RT alone in patients with locally advanced HNC. This was the specific treatment comparison required for the analysis. The Bonner trial included a mixed population with respect to cisplatin eligibility and was not specifically conducted in cisplatin-ineligible patients. However, no phase III trial was identified that evaluated RT + CTX versus RT alone specifically in cisplatin-ineligible patients. Subsequent phase III trials primarily compared RT + CTX with cisplatin-based chemoradiotherapy and therefore did not provide the comparator required for our analysis. The mixed population in the Bonner trial and the applicability of its findings to the intended cisplatin-ineligible population have therefore been acknowledged as a limitation.

For *Analysis II*, the EXTREME trial was selected because it provided phase III randomized evidence evaluating cetuximab-based chemotherapy in R/MHNC and reported the survival estimates required for the model. TPExtreme is another cetuximab-and platinum-based chemotherapy regimen used for R/MHNC; however, evidence for this regimen is derived from a phase II trial and is not considered a category 1 recommendation as per National Comprehensive Cancer Network (NCCN) guidelines. Furthermore, clinical effectiveness data for CTX-based treatment in R/MHNC from India remain limited and are primarily based on small retrospective studies, with chemotherapy selection varying according to treating physician preference (Bahl et al., 2020; Rangaraju et al., 2012). Given the higher level of evidence from a randomized phase III trial and the use of a standardized treatment protocol, the EXTREME trial was considered the most appropriate source of clinical effectiveness data for the present analysis.

The published KM curves for locoregional control and PFS given in respective trials were digitized using Engauge Digitizer version 4.1 (Engauge Digitizer, 2022). The digitized survival probabilities, together with the reported numbers at risk and available event information, were used to reconstruct approximate patient-level time-to-event data using a published KM reconstruction approach. The reconstructed patient-level data were pooled for each treatment group, and parametric survival models were fitted using exponential, Weibull, log-logistic, log-normal, generalized gamma, and Gompertz distributions (Supplementary null appendix, Supplementary Figures S1 and S2). The most appropriate distribution was selected based on statistical fit, assessed using Akaike Information Criterion (AIC) and Bayesian Information Criterion (BIC), visual comparison of the fitted survival curve with the published KM curve, and clinical plausibility of the long-term extrapolation (Latimer, 2013). The selected survival functions were used to estimate monthly transition probabilities from the initial PFS1 state to LRR health state *for Analysis I* and monthly probabilities to be in PFS2 health state for *Analysis II* (Table 1). We used cumulative incidence of distant metastasis to compute monthly transition probability to move from PFS1 to DM and LRR to DM (Bonner et al., 2006), assuming 10% patients moving from PFS1 to DM and the remaining 90% from LRR to DM.

**TABLE 1:** Model input parameters for effectiveness analysis across treatment strategies for locally advanced and recurrent/metastatic head and neck cancer in India.

Input parameter	Type of input parameter	RT alone	RT + cetuximab	Platinum-based chemotherapy alone	Platinum-based chemotherapy + cetuximab	Model specification	PSA distribution	Source
Monthly state transition probabilities								
PFS1 to LRR*	Time-varying	0.013–0.043	0.011–0.028	NA	NA	Parametric survival model; Lognormal	Beta	Bonner et al. (Bonner et al., 2006)
PFS1 to DM	Constant	0.00154	0.00072	NA	NA	-	Beta	Bonner et al. (Bonner et al., 2006)
LRR to DM	Constant	0.0061	0.0065	NA	NA	-	Beta	Bonner et al. (Bonner et al., 2006)
PFS2 to PFS2*	Time-varying	NA	NA	0.728–0.998	0.840–0.998	Parametric survival model; Log-logistic	Beta	EXTREME Trial (Vermorken et al., 2014)
PD to Death due to cancer	Constant	0.0196	0.0196	0.0196	0.0196	-	Beta	Nandakumar et al., 2016 (Bahl et al., 2020)
Utilities (Stage III–IV Head and Neck Cancer)								
PFS	Fixed	0.603 (0.422–0.783)	0.603 (0.422–0.783)	0.603 (0.422–0.783)	0.603 (0.422–0.783)	-	Beta	Dixit J et al. (Latimer, 2013)
LRR/DM	Fixed	0.458 (0.320–0.595)	0.458 (0.320–0.595)	0.458 (0.320–0.595)	0.458 (0.320–0.595)	-	Beta	Dixit J et al. (Latimer, 2013)

* Time-varying parameters represent the minimum and maximum monthly probabilities across model cycles, derived from parametric survival models.

PFS- progression free survival; PD, Progressive disease; LRR- locoregional recurrence, DM-Distant metastasis; PSA, Probabilistic sensitivity analysis; NA, Not applicable.

We derived disease-specific mortality rates using the 5-year survival estimates reported by Nandakumar et al. (Nandakumar and Nandakumar, 2016). The study provided 5-year survival estimates stratified by cancer site, disease stage, and treatment. For our analysis, we used the estimates for locally advanced disease, as this corresponds to the disease stage represented in our model population. The 5-year survival estimates for oropharyngeal, hypopharyngeal, and laryngeal cancers were weighted according to the number of patients at each cancer site, resulting in an overall 5-year survival estimate of approximately 30.4% for the cancer-site distribution used in the model. The corresponding 5-year mortality estimate was converted to an annual and subsequently monthly mortality probability using standard methods. These monthly probabilities of disease-specific mortality were then applied to the LRR and DM health states in the model. The same disease-specific mortality probabilities were applied to both the intervention and comparator arms. Age specific all-cause mortality rates were obtained from the Indian Sample Registration System (SRS) lifetables (Registrar General & Census Commissioner of India, 2014) (Supplementary Table S7).

The outcomes were assessed in terms of life-years (LYs) and quality-adjusted life years (QALYs). Stage wise utility scores were obtained from the nationally representative study (CaDCQoL) undertaken to develop a database of costs and health-related quality of life (HRQoL) (Prinja et al., 2021; Dixit et al., 2024). As part of the CaDCQoL study, primary data were collected from 1,113 head and neck cancer patients selected from 6 Indian states, who were interviewed using EQ-5D-5L tool (Table 1). The Indian tariff values were used to calculate the index utility scores (Jyani et al., 2022). We analysed this primary data to determine health state specific utility weights.

### Cost of treatment of LAHNC and R/MHNC

The costs were estimated from an abridged societal perspective for all treatment arms. In *Analysis I and II*, the cost of treatment in the PFS, LRR, PD and DM state included the cost of outpatient consultations, diagnostics such as endoscopy and biopsy, radiology and laboratory investigations. In addition, in *Analysis I*, costs for patients in PFS state included consultation for dental evaluation, nutrition/speech, cetuximab, RT, management of adverse effects (grades 3–4), and routine follow-up (Table 2; Supplementary null Appendix; Supplementary Tables S1 and S2). The additional costs for treatment in LRR health state included cost of administration of systemic agents (Gupta et al., 2021), surgery, and RT. In DM state, the cost for administration of first-line and second-line systemic agents and maintenance therapy were additionally included. Different systemic regimens (platinum-based chemotherapy, taxanes, immune check point inhibitors or targeted therapy given alone or in combination with chemotherapy, oral or injectable metronomic therapy) were administered as part of first line therapy. For patients with PD after first line systemic therapy, the second line therapy included best supportive care, metronomic therapy, single agent chemotherapy including platinum or taxanes, etc. (Supplementary Tables S3-S4).

**TABLE 2:** Cost parameters for assessing the cost-effectiveness of Cetuximab for the treatment of LAHNC and R/M HNC in India.

Treatment parameters	Cost estimate (₹)	PSA distribution	Source
Radiotherapy RT (70 Gy/35 fractions/7 weeks)			
Intensity-Modulated Radiation Therapy (IMRT)	70,000	Gamma	HBP 2022 (Health Benefit Package, 2022)
Three-Dimensional Conformal Radiation Therapy (3DRT)	21,000	Gamma	HBP 2022 (Health Benefit Package, 2022)
Two-Dimensional Conformal Radiation Therapy (2DRT)	11,000	Gamma	HBP 2022 (Health Benefit Package, 2022)
Chemotherapy			
Health System cost per day-care visit	1,038	​	Gupta N et al. (Gupta et al., 2021)
Injection Cetuximab (per 100 mg vial) – Original	11,000	Gamma	Market price (IndiaMART, 2026a)
Injection Cetuximab (per 100 mg vial) – Biosimilar	6,500	Gamma	Market price (IndiaMART, 2026b)
*Surgery cost*			
*Neck dissection/local Sx*	26,579	Gamma	HBP 2022 (Health Benefit Package, 2022)
Other chemotherapeutic drug regimens			
Paclitaxel (175 mg/m^2^) + Carboplatin (AUC 5) every 21 days	14,500	Gamma	HBP 2022 (Health Benefit Package, 2022)
Paclitaxel 175 mg/m^2^ every 21 days	11,800	Gamma	HBP 2022 (Health Benefit Package, 2022)
Inj Cisplatin 100 mg/m^2^ every 21 days	9,800	Gamma	HBP 2022 (Health Benefit Package, 2022)
Injection Pembrolizumab 200 mg IV	150,000	Gamma	Market price (Tata 1mg, 2026)
Injection Nivolumab 100 mg vial	50,000	Gamma	Market price (CUB Pharmacy, 2026)
Injection Carboplatin AUC 5	7,000	Gamma	HBP 2022 (Health Benefit Package, 2022)
Injection Docetaxel 75 mg/m^2^ every 21 days	8500	Gamma	HBP 2022 (Health Benefit Package, 2022)

HBP, Health benefit package; PSA, Probabilistic sensitivity analysis; AUC, Area under the curve; Gy, Gray.

For *Analysis II*, additional costs for PFS included costs of maintenance cetuximab, management of adverse effects and follow up. For patients in PD state, costs for second-line systemic agents, including taxanes, immune check point inhibitors or targeted therapy given alone or in combination with chemotherapy, oral or injectable metronomic therapy, and best supportive care including palliative RT were included. The costs were applied separately in each cycle using the treatment protocol obtained from the standard treatment guidelines as per Indian Council of Medical Research (ICMR) supplemented by expert opinion (Prabhash et al., 2020). (Supplementary Tables S3-S4).

We used reimbursement rates under the PM-JAY scheme, India’s national insurance program to elicit the cost of RT and chemotherapeutic regimens as shown in Table 2 (Health Benefit Package, 2022). The reimbursement rates are inclusive of chemotherapeutic agents, recurring investigations, day care/inpatient charges, supportive care and professional charges. We also included the direct non-medical expenditure (including travelling, boarding/lodging, food, informal payments, etc.) based on analysis of data from the CADCQoL database (Prinja et al., 2021) (Prinja et al., 2023b).

For cetuximab and certain other immunotherapy agents like injection pembrolizumab, injection nivolumab, *etc.*, which were not included under any publicly financed health insurance scheme, we used market prices (IndiaMART, 2026a; IndiaMART, 2026b; Tata 1mg, 2026; CUB Pharmacy, 2026) (Table 2). For estimating the cost of Cetuximab, we considered a 100-mg vial of drug. Based on an assumed BSA of 1.6 m^2^, the loading dose of 400 mg/m^2^ corresponded to 640 mg, requiring seven 100-mg vials. As vial sharing was not assumed, the cost of all seven vials was included, with the remaining 60 mg considered as wastage. For the subsequent weekly administrations of Cetuximab in a dosage of 250 mg/m^2^, the dose corresponded to 400 mg at a BSA of 1.6 m^2^, for which the cost of four 100-mg vials was included per administration. To account for the cost of diagnostic services, we used the provider payment rates under the Central Government Health Scheme (CGHS) – a publicly financed national insurance scheme (CGHS, 2020). All costs are reported in Indian National Rupee (₹) and converted to United States Dollar ($) using an exchange rate of 1$ = ₹ 91.6 (XE.com, 2026).

We also accounted for Cost for management of commonly occurring (>5%) grade 3–4 adverse events in both the analyses. In case of skin reactions with cetuximab, we included cost of managing grade 2–4 toxicity. The percentage of commonly reported adverse events considered in both *Analysis I and II* are depicted in Supplementary Table S5, S6 respectively. The data on incidence of adverse events were obtained from respective trials. All costs in the analysis are expressed in 2024–25 Indian National Rupees (INR). Costs obtained from the Health Benefits Package (HBP) 2022 and CGHS (Cost of diagnostics) were retained at the reported package rates because these package prices remained unchanged and applicable during 2024–25. Therefore, no inflation adjustment was applied to these HBP package costs. Costs obtained from market sources, including cetuximab, injection pembrolizumab, and injection nivolumab were based on the corresponding 2024–25 market prices. Thus, all cost inputs were standardized to the same year (2024–25).

### Sensitivity analysis

Incremental cost per QALY gained (ICER) with use of cetuximab in both *Analysis I and II* were computed. We used the published Indian cost-effectiveness threshold (CET) of one-time *per capita* gross domestic product (GDP) to assess the cost-effectiveness, corresponding to CET of ₹247,045 (US$2,697) for 2024–25 (Chugh et al., 2026; World Bank, 2024). A multivariable probabilistic sensitivity analysis (PSA) was undertaken to estimate the effect of joint parameter uncertainty (Doubilet et al., 1985). Under PSA, all cost parameters were assigned gamma distribution, while utility values and probabilities/proportions were assigned beta distribution. The value of standard error (SE) was used to create a distribution around the point estimate of a parameter. In cases where SE was not reported, a variation of 50% and 10% on either side of the base value was used for cost and clinical parameters respectively. The median value of incremental cost-effectiveness ratio (ICER) along the 2.5th and 97.5th percentile was calculated using 999 Monte Carlo simulations. In addition, we also evaluated alternative survival distributions to assess the uncertainty associated with the choice of parametric distribution.

## Ethical approval

Ethical approval was obtained from Institutional Ethics Committee of Post Graduate Institute of Medical Education and Research, India with reference number IEC-03/20,202–1,565. The present cost-effectiveness analysis did not involve recruitment of participants or collection of new primary data. Health-state utility weights and out-of-pocket expenditure estimates were derived from primary data collected previously as part of the CaDCQoL study, which included 1,113 patients with head and neck cancer from six Indian states. Participants provided written informed consent as part of the original CaDCQoL study, which received the required ethical approval. The present analysis used anonymised data from the CaDCQoL study for the purpose of data analysis. No additional primary data collection was undertaken for the present study.

## Results

In *Analysis I*, we estimated that a patient having LAHNC incurs a lifetime cost of ₹ 683,036 (95% CI: ₹ 525,678- ₹ 846,675) when treated with RT alone as compared to ₹ 921,014 (95% CI: ₹ 758,124–1,124,327) and ₹ 821,584 (95% CI: ₹ 661,283 - ₹ 980,358) when treated with RT + original or biosimilar CTX respectively. The life years lived with RT alone and RT + CTX were found to 4.64 (95% CI: 4.29–4.99) and 5.01 (95% CI: 4.71–5.30) respectively. This translated into 2.45 (95% CI: 1.92–2.94) (RT alone) and 2.73 (95% CI: 2.11–3.30) (RT + CTX) QALYs in the two arms (Table 3).

**TABLE 3:** Base-case cost-effectiveness outcomes for radiotherapy alone compared with radiotherapy plus Original and biosimilar cetuximab for locally advanced head and neck cancer in India.

Parameter	Radiotherapy (RT) alone	RT + biosimilar cetuximab	RT + original cetuximab
Per-person life-years (LYs) lived	4.64 (4.29–4.99)	5.01 (4.71–5.30)	5.01 (4.71–5.30)
Per-person quality-adjusted life-years (QALYs) lived	2.45 (1.92–2.94)	2.73 (2.11–3.30)	2.73 (2.11–3.30)
Per-person lifetime costs (₹)	683,036 (525,678–846,675)	821,584 (661,283–980,358)	921,014 (758,124–1,124,327)
Incremental LYs gained	—	0.37	0.37
Incremental QALYs gained	—	0.28	0.28
Incremental cost (₹)	—	138,548	237,978
Incremental cost per LY gained	—	358,503	639,296
Incremental cost per QALY gained	—	501,320	871,632

In *Analysis II*, the lifetime cost incurred by a R/MHNC patient was estimated to be ₹ 200,624 (95% CI: ₹ 151,935-₹ 261,829) when treated with platinum-based CT as against ₹ 1,240,951 (95% CI: ₹ 779,346- ₹ 2,035,898) and ₹ 811,400 (95% CI: ₹ 531,650- ₹ 1,249,887) when treated with CT + original or biosimilar CTX respectively. The life years lived with CT alone and CT + CTX were found to be 1.50 (95% CI: 1.48–1.51) and 1.53 (95% CI: 1.50–1.56) respectively. After applying the utility values, this translates into 0.74 (95% CI: 0.57–0.90) (CT alone) and 0.78 (95% CI: 0.61–0.96) (CT + CTX) QALYs respectively (Table 4). Detailed stratified treatment costs by health state and treatment strategy are presented in Table 5.

**TABLE 4:** Base-case cost-effectiveness outcomes for Platinum-based chemotherapy alone compared with Platinum-based chemotherapy plus Original and biosimilar cetuximab for recurrent/metastatic squamous cell carcinoma of head and neck in India.

Parameter	Platinum-based chemotherapy	Platinum-based chemotherapy + biosimilar cetuximab	Platinum-based chemotherapy + original cetuximab
Per-person life-years (LYs) lived	1.50 (1.48–1.51)	1.53 (1.50–1.56)	1.53 (1.50–1.56)
Per-person quality-adjusted life-years (QALYs) lived	0.74 (0.57–0.90)	0.78 (0.61–0.96)	0.78 (0.61–0.96)
Per-person lifetime costs (₹)	200,624 (151,935–261,829)	811,400 (531,650–1,249,887)	1,240,951 (779,346–2,035,898)
Incremental LYs gained	—	0.03	0.03
Incremental QALYs gained	—	0.04	0.04
Incremental cost (₹)	—	610,776	10,40,327
Incremental cost per LY gained	—	16,709,547	27,924,496
Incremental cost per QALY gained	—	15,023,037	24,693,072

**TABLE 5:** Stratified Costs of Treatment by Health State and Treatment strategy.

Cost component	RT + cetuximab (₹)	RT alone (₹)
Total cost during the treatment in the Progression-free survival (PFS) health state	**661,944**	**413,449**
Diagnostic work-up	10,395	10,395
Outpatient consultation (including Ryle’s tube insertion cost)	468	468
Laboratory tests	7,632	7,632
Cetuximab cost, including pre- and post-medications	226,851	—
Radiotherapy	338,800	338,800
Response assessment at 6 and 12 weeks after CT-RT and follow-up costs	60,568	48,118
Adverse event management	17,230	8,036
Total cost following locoregional recurrence (LRR)	**220,399**	**252,627**
Total cost following distant metastasis (DM)	**40,344**	**49,008**
Total undiscounted cost	**922,687**	**715,084**
Total discounted cost	**891,100**	**682,139**
Cost component	Chemotherapy + Cetuximab (₹)	Chemotherapy alone (₹)
Total cost during the treatment in the PFS health state	**859,205**	**132,135**
Diagnostic work-up	1,070	1,070
Laboratory tests	7,094	2,717
Other diagnostic tests (CECT, PET, MRI)	19,601	14,102
Chemotherapy cost	40,036	40,036
Cetuximab cost	44,851	0
Maintenance therapy	682,501	—
Adverse event management cost	30,382	35,041
Progressive disease (PD) cost	**95,995**	**111,491**
Total undiscounted cost	**921,530**	**204,456**
Discounted total cost	**908,932**	**201,257**

Abbreviations: CECT, Contrast-Enhanced Computed Tomography; PET, Positron Emission Tomography; MRI, Magnetic Resonance Imaging.

These cost estimates represent deterministic findings of the present analysis. All bold values represent total costs (health-state specific and overall costs).

### Cost-effectiveness

In *Analysis I*, the incremental cost per QALY gained with use of RT alone with original or biosimilar CTX was estimated as ₹ 871,632 and ₹ 501,320 respectively. Similarly in *Analysis II*, the incremental cost incurred per QALY gained with use of RT + CTX was estimated as ₹ 24,693,072 and ₹ 15,023,037 with original and biosimilar molecule respectively.

At a WTP threshold of one-time *per capita* GDP of India, i.e., ₹ 247,045, the RT + CTX (biosimilar) becomes cost-effective when the price of biosimilar CTX is reduced by 50%. At this price, the median ICER is ₹ 222,239, and the probability of being cost-effective is 64.9%. With further price reductions, the probability becomes higher, reaching 87.3% at a 60% reduction in drug price (Figure 3).

**FIGURE 3:**
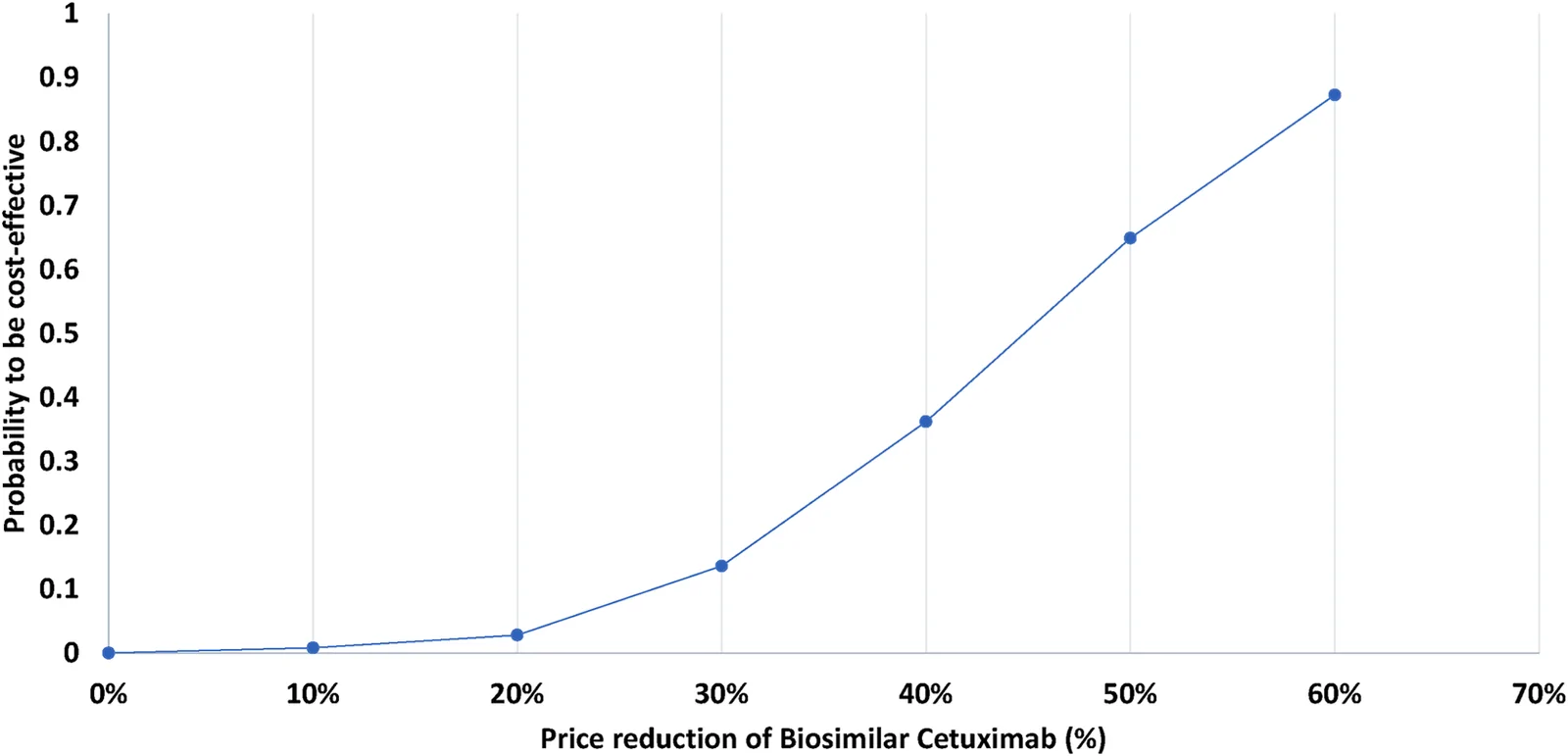
Probability of Biosimilar Cetuximab to be cost-effective for the treatment of locally advanced squamous cell carcinoma of head and neck, at different levels of price reduction.

### Scenario analysis

The choice of parametric survival distribution had a limited impact on the cost-effectiveness results in *Analysis I*. Compared with the Log-normal distribution (selected based on the lowest AIC/BIC and visual assessment of model fit; median ICER: ₹501,320/QALY) used in *Analysis I*, the median ICER increased by 9.2% with the Weibull distribution (₹547,441/QALY), 4.1% with the Exponential distribution (₹521,874/QALY), and 6.8% with the Log-logistic distribution (₹535,410/QALY). In contrast, the use of the Gompertz distribution resulted in a 8.1% decrease in the median ICER (₹460,713/QALY). Importantly, these variations did not change the overall direction of the results, with the intervention remaining not cost-effective at a WTP of one-times *per capita* GDP across all alternative survival distributions.

A similar assessment in *Analysis II* showed an increase in the estimated ICER across alternative survival distributions. Compared with the Log-logistic distribution (median ICER: ₹15,023,037/QALY), all alternative distributions resulted in higher median ICERs. The ICER value increased by 35.8% with the Log-normal distribution (₹20,401,284/QALY), 23% with the Exponential distribution (₹18,478,335/QALY), 27.1% with the Weibull distribution (₹19,094,280/QALY), and 22% with the Gompertz distribution (₹18,328,105/QALY). Nevertheless, these variations did not alter the overall conclusion, with the intervention remaining not cost-effective at currently accepted WTP threshold for India, across all alternative survival distributions.

## Discussion

At the current price, use of biosimilar cetuximab incurs an incremental cost per QALY gained which is 2.02 and 60.8 times the *per capita* GDP of India, in the two evaluated scenarios viz, *Analysis I* and *Analysis II*, respectively. Hence, cetuximab is not a cost-effective treatment option for patients with LAHNC and R/MHNC patients in India at the current price and recommended WTP threshold. However, a price reduction of 50% is likely to make use of biosimilar cetuximab cost-effective for treating cisplatin-ineligible LAHNC patients in India.

RT + CTX was found to be a cost-effective alternative to RT alone for LAHNC patients in five European countries (Belgium, Italy, France, Switzerland and United Kingdom) with incremental costs per QALY ranging from €7538 to €10,836 (Brown et al., 2008). The combination was also found to be cost effective in Taiwan (Chan et al., 2011) and the Netherlands (van et al., 2015). The ICER for RT + CTX in our study is 2 times the *per capita* GDP of India. The studies that found RT + CTX to be a cost-effective alternative to RT used a threshold of 3 times their respective *per capita* GDP, which if used in our study, will also make RT + CTX a cost-effective option for India.

Currently, immune check point inhibitors used alone or in combination with platinum-based CT are preferred first line option for patients with R/MHNC (National Comprehensive Cancer Network, 2024). However, pembrolizumab is inaccessible to majority of patients in India due to the high costs. In a retrospective audit from Tata Memorial Hospital Mumbai, of the 2,796 platinum-refractory HNSCC patients eligible for immunotherapy, only 41 (1.47%) patients received immunotherapy due to cost and financial un-affordability (Patil et al., 2021). Immunotherapy based treatment is also less effective in oral cavity tumours, which forms the most common sub-group in HNCs in India (Bagal et al., 2023; Hobday et al., 2022). With the availability of biosimilar of cetuximab, it becomes one of the preferred options for R/MHNCs in India.

Platinum based CT + CTX compared to CT alone for R/MHNC patients was not found to be cost effective in previous analysis done in China (ICER $172,702/QALY; incremental QALY 0.138) (Lang and Dong, 2020), Canada (ICER CaD $386,000/QALY; incremental QALY 0.093) (Hannouf et al., 2012) and the United Kingdom ((ICER 121,367/QALY; incremental QALY 0.187) (Greenhalgh et al., 2009). All these ICERs were above the country-specific threshold for cost-effectiveness. This is largely attributable to marginal benefits in the QALYs with the addition of cetuximab to platinum-based CT. As a result, the combination fails to be a cost-effective option even in high income countries. Further research for specific subgroups like patients with combined positive score (CPS) < 1 and oral cavity cancer in India, may better define the role of cetuximab in R/MHNCs. Besides the EXTREME protocol, TPExtreme is another preferred protocol with cetuximab and platinum-based chemotherapy used in India (Guigay et al., 2021). In our analysis, the cost of cetuximab was the major determinant of the incremental costs associated with cetuximab-based treatment. The survival benefits and dose of cetuximab used in TPExtreme is similar to EXTREME protocol and hence is unlikely to be cost-effective.

### Model validation

We also validated our model with existing published literature. We found that our model findings on the rate of locoregional control in RT alone arm at 1-year (59.6%), 2-years (38.9%) and 3-years (27.5%) was in line with the published literature (Bonner et al., 2006). Similarly, the rate of locoregional control in RT + CTX arm at 1-year (71.2%), 2-years (50%) and 3-years (47%) was in line with the published literature (Bonner et al., 2006). Further, we found that the risk of loco-regional recurrence reduced by 27.2% with use of RT + CTX, which is consistent with reported 32% risk reduction in local-regional recurrence with use of cetuximab along with RT (Bonner et al., 2006). Five-year survival with radiotherapy (RT) alone was found to be 50.9%, which is again consistent with previously reported estimates ranging from 50% (Goyal et al., 2025) to 56.45% (Lakshmi et al., 2025), with RT as the main treatment modality, among patients with LAHNC in India (Table 6).

**TABLE 6:** Comparison of modelled clinical outcomes with published evidence for radiotherapy (RT) alone and RT plus cetuximab.

Effectiveness parameter	Radiotherapy alone		Radiotherapy + cetuximab		Source of published evidence
​	Present model	Published evidence	Present model	Published evidence	​
Loco-regional control (LRC)					
1-year LRC	59.6%	55%	71.2%	63%	Bonner et al., 2006 (Bonner et al., 2006)
1-year DFS	-	-	71.2%	85%	Rawat et al., 2020 (Rawat et al., 2020)
2-year LRC	38.9%	41%	51.7%	50%	Bonner et al., 2006 (Bonner et al., 2006)
2-year DFS	​	​	51.7%	69%	Rawat et al., 2020 (Rawat et al., 2020)
3-year LRC	27.5%	34%	39.4%	47%	Bonner et al., 2006 (Bonner et al., 2006)
Median duration (in months)	16	14.9	25	24.4	Bonner et al., 2006 (Bonner et al., 2006)
Risk of loco-regional recurrence (LRR)					
Risk reduction at 12 months	​	​	27.2%	32%	Bonner et al., 2006 (Bonner et al., 2006)
Overall survival					
5-year survival	50.9%	48.3% (larynx)	61.7%	48.3% (larynx)	Nandakumar et al., 2016 (Nandakumar and Nandakumar, 2016)
Median OS (in months)	-	-	78	65 ± 18	Tryambake et al., 2025 (Tryambake et al., 2025)
Reduction in risk of death	-	-	20%	26%	Bonner et al., 2006 (Bonner et al., 2006)

For external validity, model-generated outcomes were compared with real-world evidence from India. Rawat et al. (2020) reported 1-year and 2-year disease-free survival of 85% and 69% (Lakshmi et al., 2025), respectively, among patients with unresectable locally advanced squamous cell carcinoma of head and neck (SCCHN) receiving RT + CTX, compared with 71.7% and 53.3% estimated in the present model. The observed difference may be partly explained by differences in case-mix and disease stage distribution between the two sources. Rawat et al. did not include patients with hypopharyngeal cancers, which are known to have comparatively poorer survival outcomes (Lakshmi et al., 2025). In contrast, the Bonner trial used in our study included hypopharyngeal cases, thereby incorporating a subgroup with worse prognosis and resulting in relatively lower survival estimates in our model. Additionally, a substantially higher proportion of patients in the Bonner trial were in stage IV (76%) compared to Rawat et al. (∼20%), thereby incorporating a population with worse prognosis and resulting in relatively lower survival estimates in the model (Bonner et al., 2006) (Rawat et al., 2020).

Similarly, Tryambake et al. (2025) reported a median overall survival of 47–83 months among the patients receiving RT + CTX in the first-line setting (Tryambake et al., 2025), compared with 78 months estimated in our model. Overall, the model outputs were broadly consistent with published literature and real-world evidence. The relatively lower health outcomes estimated in the model are likely owing to differences in case-mix and disease severity particularly the inclusion of hypopharyngeal cancers and a higher proportion of stage IV patients in the Bonner trial (Bonner et al., 2006).

Further, model-generated survival and health outcomes for *Analysis I and II* were compared with those reported in published economic evaluations as shown in Table 7. The present model findings (*Analysis I and II*) are broadly consistent with published evidence as shown in Table 7. Across studies pertaining to *Analysis I*, the addition of cetuximab to radiotherapy demonstrated a consistent improvement in survival and quality-adjusted survival, with incremental QALY gains ranging from 0.24 to 1.23 (Brown et al., 2008) (van et al., 2015) (Griffin et al., 2009) (Cabellos et al., 2025). The incremental gain of 0.28 QALYs estimated in the present analysis lies within this range. Differences in absolute outcomes and magnitude of benefit can be explained by variations in modelling approaches and assumptions across studies. For instance, Griffin et al. (2009) and Linden et al. (van et al., 2015) employed mixture cure models, which typically yield higher long-term survival estimates and consequently higher QALYs compared to conventional survival extrapolation methods (Latimer, 2013). The present analysis employed conventional parametric survival extrapolation methods rather than mixture cure models, as the latter require strong assumptions regarding the presence of a “cured” fraction of patients. In head and neck cancers, particularly in recurrent or metastatic settings, long-term cure is generally uncommon, and survival data are often insufficiently mature to robustly estimate a cured proportion. In such circumstances, mixture cure models may overestimate long-term survival and QALYs, leading to potentially optimistic cost-effectiveness results. Conventional survival models, by contrast, provide a more conservative and empirically grounded approach when the evidence for cure is limited. This approach is consistent with recommendations from the National Institute for Health and Care Excellence Decision Support Unit, which advises that mixture cure models should only be used when there is strong clinical and statistical evidence supporting a cure fraction (National Institute for Health and Care Excellence Decision Support Unit, 2013).

**TABLE 7:** Comparison of model-based survival and utility outcomes with published economic evaluation evidence.

Outcome parameter	Comparator		Intervention		Published evidence
Absolute survival outcomes					
Analysis I	RT		RT + CTX		​
​	Present model	Published evidence	Present model	Published evidence	Source of published evidence
LYs	4.64 (4.29–4.99)	4.18	5.01 (4.71–5.30)	5.10	Brown et al., 2008 (Brown et al., 2008)
QALYs	2.45 (1.92–2.94)	2.88	2.73 (2.11–3.30)	3.96	Brown et al., 2008 (Brown et al., 2008)
		2.81		3.85	Griffin et al., 2007 (Griffin et al., 2009)
		1.12		1.36	Cabellos RÁ et al., 2025 (Cabellos et al., 2025)
		5.737		6.962	Linden et al., 2015 (van et al., 2015)
Analysis II	CT		CTX + CT		​
​	Present model	Published evidence	Present model	Published evidence	Source of published evidence
LYs	1.50 (1.48–1.51)	1.02	1.53 (1.50–1.56)	1.26	Lang et al., 2020 (Lang and Dong, 2020)
		-		1.32	Liu et al., 2025 (Liu et al., 2025)
QALYs	0.74 (0.57–0.90)	0.53	0.78 (0.61–0.96)	0.669	Lang et al., 2020 (Lang and Dong, 2020)
		-		1.00	Liu et al., 2025 (Liu et al., 2025)

Abbreviations: RT- radiotherapy, CTX- cetuximab, CT- Platinum-based Chemotherapy, LYs, Life years, QALYs- Quality-adjusted life years.

Additionally, differences in discounting rates may have influenced the results; Brown et al. (Brown et al., 2008) applied a higher discount rate of 3.5% to both costs and health outcomes, thereby reducing the present value of health gains, which may partly explain the lower life years reported for both RT alone (4.18 vs. 4.64 [4.29–4.99]) and RT + CTX (5.10 vs. 5.01 [4.71–5.30]) compared with the present model. In contrast, QALYs reported in Brown et al. were higher for both RT alone (2.88 vs. 2.45 [1.92–2.94]) and RT + CTX (3.96 vs. 2.73 [2.11–3.30]) relative to our findings, primarily due to the use of substantially higher utility values for the post-acute loco-regional control health state (0.862 vs. 0.603 [0.422–0.783]) in the present model. Secondly, Cabellos et al. adopted a shorter 5-year time horizon, which likely resulted in lower QALY estimates due to limited capture of long-term survival benefits (Cabellos et al., 2025).

In Analysis II (chemotherapy-based regimens), the LYs and QALYs gained in our study were higher compared with those reported by Lang et al. (2020) (Lang and Dong, 2020) and Liu et al. (2025) (Liu et al., 2025). This is primarily because we used effectiveness parameters from the EXTREME trial (Vermorken et al., 2014), which demonstrated a slightly greater treatment benefit with cetuximab plus chemotherapy (CT + CTX), reporting a hazard ratio (HR) of 0.54 (46% reduction in hazard) compared with an HR of 0.57 (43% reduction in hazard) derived from the CHANGE-2 trial used in the other two studies (GuoY et al., 2021).

Similarly, Hannouf et al. (2012) reported higher incremental health gains of 0.136 LYs and 0.093 QALYs (Hannouf et al., 2012), whereas we estimated incremental gains of 0.03 LYs and 0.04 QALYs. The higher incremental LY gain may be explained by differences in the mortality assumptions between the two studies. Hannouf et al. derived mortality from the EXTREME trial; however, the probability of death used in their model was not explicitly reported in the published paper. For comparison, we calculated the monthly probability of death from the median OS reported in the EXTREME trial, which was 0.090 in the chemotherapy arm and 0.066 in the chemotherapy plus cetuximab arm, compared with 0.196 (derived using survival estimates reported in an Indian study by Nandakumar et al., 2016) used in our model (Nandakumar and Nandakumar, 2016). The higher mortality in our model may have contributed to the lower incremental LY gain. Further, the lower incremental QALY gain is due to the use of lower utility values for progression-free survival (PFS: 0.603) and progressive disease (PD: 0.458) compared with those used by Hannouf et al. (2012) (PFS: 0.65; PD: 0.52) (Hannouf et al., 2012).

## Policy implications

Our analysis does not support the inclusion of cetuximab at current market price in the HBPs for the treatment of LAHNC or RMHNC patients. However, a 50% price reduction for the biosimilar cetuximab results in 64.9% probability of cetuximab to be a cost-effective option for use with RT for patients of LAHNCs who are ineligible for cisplatin. This calls for price negotiation as part of broader strategic purchasing arrangements if cetuximab is to be included in the health benefits package.

### Limitations

There are certain limitations of our analysis. Firstly, we have used the international evidence for deriving transition probabilities for both intervention and control arms, i.e., movement of patients from PFS1 to LRR, PFS1 to DM1 in *analysis I* (Bonner et al., 2006) and probability to remain in PFS2 health state in *analysis II* (Vermorken et al., 2008). However, there are no corresponding robust country-specific estimates, and majority previous CEAs have derived estimates from the same sources. Secondly, the effectiveness estimates in *Analysis I* were not derived from evidence specifically generated in a cisplatin-ineligible population. In the absence of randomized evidence directly comparing RT + CTX with RT alone exclusively in cisplatin-ineligible patients, the Bonner trial represented the best available direct evidence. Thirdly, the selection of systemic therapies in *Analysis II* may not fully reflect the evolving treatment landscape for recurrent or metastatic head and neck cancer. Although both pembrolizumab and cetuximab-based regimens are Category 1 options in the NCCN guidelines, we did not include pembrolizumab based regimens as the policy question framed by the National Health Authority for inclusion in PM-JAY health benefit package was specifically related to cetuximab and did not refer to pembrolizumab. Fourthly, we have not considered the cost of grade 1–2 adverse events which might have slightly underestimated the costs. Lastly, we did not consider the indirect costs due to loss of productivity incurred by the patients as well as the caregivers. This was in agreement with Indian HTA guidelines (HTAIn, 2021).

## Conclusion

Our study supports the evidence that cetuximab improves survival and quality of life among LAHNC as well as R/MHNC patients. However, at the current price, the addition of cetuximab to radiotherapy and platinum-based chemotherapy does not represent a good value for money for treatment of patients with LAHNC and R/MHNC respectively. A price reduction of 50% would be required to make biosimilar cetuximab cost-effective for treating LAHNC patients in India. For R/MHNC, cetuximab is unlikely to be cost-effective at its current price given the substantial difference between its current price and the WTP threshold. Nevertheless, further research is warranted to better characterise its value in clinically relevant patient subgroups. In particular, more robust evidence on the effectiveness and cost-effectiveness of cetuximab among patients with CPS <1, and those with oral cavity subsites would be valuable, as the EXTREME trial used in our analysis provides survival estimates for the overall R/MHNC population rather than these specific subgroups. Such evidence could help identify patient populations most likely to benefit from cetuximab and support its more targeted and efficient use.

## Data Availability

The original contributions presented in the study are included in the article/Supplementary Material, further inquiries can be directed to the corresponding author.
