# Supplementary material for: Cost-effectiveness analysis of cetuximab for the treatment of head and neck cancers in India
Source: Front Pharmacol. 2026 Oct 23;17:1948615. doi: 10.3389/fphar.2026.1948615 (PMC13739782; doi:10.3389/fphar.2026.1948615)
Supplement: Supplementary file 1 [file Supplementaryfile1.docx]

**Supplementary Appendix**

**Table of Contents**

[**Table S1: Cost parameters for assessing the cost-effectiveness of Radiotherapy with Concomitant Cetuximab and Radiotherapy alone**	3](#_Toc239674226)

[**Table S2: Cost parameters used for assessing the cost-effectiveness of Platinum-based chemotherapy combined with Cetuximab and Platinum-based chemotherapy alone**	3](#_Toc239674227)

[**Table S3: Proportions used for assessing the cost-effectiveness of Radiotherapy with**	4](#_Toc239674228)

[**Concomitant Cetuximab and Radiotherapy alone**	4](#_Toc239674229)

[**Table S4: Proportions used for assessing the assessing the cost-effectiveness of Platinum-based chemotherapy combined with Cetuximab and Platinum-based chemotherapy alone**	5](#_Toc239674230)

[**Table S5: Common adverse events considered for cost-effectiveness analysis of RT alone and RT combined with Cetuximab**	5](#_Toc239674231)

[**Table S6: Common adverse events considered for cost-effectiveness analysis of platinum-based chemotherapy alone and chemotherapy combined with Cetuximab**	6](#_Toc239674232)

[**Table S7: Age-Specific Monthly Probability of Mortality from Natural Causes**	6](#_Toc239674233)

[**Figure S1 (a): Loco-regional control curve - RT alone**	7](#_Toc239674234)

[**Figure S1 (b) Loco-regional control curve - RT plus Cetuximab**	7](#_Toc239674235)

[**Figure S2 (a) Progression free survival curve – Chemotherapy alone**	8](#_Toc239674236)

[**Figure S2 (b) Progression free survival – Chemotherapy plus cetuximab**	8](#_Toc239674237)

# **Table S1: Cost parameters for assessing the cost-effectiveness of Radiotherapy with Concomitant Cetuximab and Radiotherapy alone**

| Treatment Parameters | Cost (NABH) | Source |
| --- | --- | --- |
| *Diagnostics and Laboratory investigations* |  |  |
| Direct Laryngoscopy & Biopsy | 5750 | CGHS |
| Contrast-enhanced computed tomography (CECT)  (BOS to T4) | 2200 | CGHS |
| Magnetic resonance imaging (MRI) with Contrast | 5750 | CGHS |
| Positron emission tomography (PET) scan | 23607 | CGHS |
| Chest X-ray | 70 | CGHS |
| Contrast-enhanced computed tomography-Chest | 2000 | CGHS |
| Dental Evaluation & Tooth Extraction | 92 | CGHS |
| Nutrition/ speech/ swallowing consultation | 350 | CGHS |
| Complete blood count | 155 | CGHS |
| Renal function tests | 259 | CGHS |
| Liver function tests | 259 | CGHS |
| Serum electrolytes | 253 | CGHS |
| Fasting blood sugar | 28 | CGHS |

# **Table S2: Cost parameters used for assessing the cost-effectiveness of Platinum-based chemotherapy combined with Cetuximab and Platinum-based chemotherapy alone**

| Treatment Parameters | Cost (NABH) | Source |
| --- | --- | --- |
| Biopsy Fine needle aspiration cytology | 720 | High-end diagnostics list HBP 2022 |
| Complete blood count | 155 | CGHS |
| Renal function tests | 259 | CGHS |
| Liver function tests | 259 | CGHS |
| Fasting blood sugar | 28 | CGHS |
| Serum electrolytes | 253 | CGHS |
| Contrast-enhanced computed tomography | 2200 | CGHS |
| Positron emission tomography (PET) scan | 23607 | CGHS |
| Magnetic resonance imaging (MRI) with Contrast | 5750 | CGHS |
| Injection Cisplatin 100mg/m2 D1 | 5600 | HBP 2022 |
| Injection Carboplatin AUC 5 D1 | 7000 | HBP 2022 |
| Injection 5FU 1000mg/m2 x D1-4 | 100 | MRP |
| Injection Cetuximab 400mg/m2 week 1 | 73000 | RMSC |
| Injection Paclitaxel 175mg/m2 | 11800 | HBP 2022 |
| Injection Docetaxel 75mg/m2 | 8500 | HBP 2022 |
| Injection Methotrexate 40mg/m2 iv | 1000 | MRP |
| Best supportive care | 8500 | HBP 2022 |

# **Table S3: Proportions used for assessing the cost-effectiveness of Radiotherapy with**

# **Concomitant Cetuximab and Radiotherapy alone**

| Parameter | Percentage |
| --- | --- |
| *Diagnostics and Laboratory investigations* |  |
| Contrast-enhanced computed tomography (CECT) (BOS to T4) | 80% |
| Magnetic resonance imaging (MRI) with Contrast | 15% |
| Positron emission tomography (PET) scan | 5% |
| Chest X-Ray | 60% |
| CECT Chest | 40% |
| Ryles Tube Insertion | 50% |
| Intensity-modulated Radiation therapy | 60% |
| 3D- Conformal radiation therapy (3DCRT) | 20% |
| 2D Radiation therapy | 20% |
| *Response assessment* |  |
| CECT | 60% |
| MRI | 10% |
| PET Scan | 30% |
| *For patients who progressed to LRR< 2 years* |  |
| Surgery | 20% |
| Chemotherapy | 70% |
| Radiation therapy (RT) | 10% |
| *For patients who progressed to LRR< 2 years* |  |
| Surgery | 20% |
| Chemotherapy | 60% |
| RT | 20% |
| *Patients in Distant Metastasis: First line chemotherapy* |  |
| Chemotherapy | 100% |
| Palliative RT | 20% |
| Paclitaxel (175mg/m2) + Carboplatin (AUC 5)/ cisplatin (100mg/m2) | 25% |
| Paclitaxel 175mg/m2 X 3 weekly Or  Injection Docetaxel 75mg/m2 x 3weekly | 20% |
| Injection Cisplatin 100mg/m2/ carboplatin AUC 5 + Injection 5FU 1000mg/m2 | 25% |
| Pem + Niv + PemCF + Cetuximab | 5% |
| Iv + oral metronomic | 15% |
| Best supportive care | 10% |
| *Second line therapy: Second line chemotherapy* |  |
| Best supportive care | 50% |
| Metronomic | 20% |
| Injection Carboplatin AUC 5/ Injection Paclitaxel 175mg/m2/ Injection Docetaxel 75mg/m2 | 30% |

# **Table S4: Proportions used for assessing the assessing the cost-effectiveness of Platinum-based chemotherapy combined with Cetuximab and Platinum-based chemotherapy alone**

| Parameters | Percentage |
| --- | --- |
| Contrast-enhanced computed tomography | 75% |
| Positron emission tomography (PET) scan | 20% |
| Magnetic resonance imaging (MRI) with Contrast | 5% |
| Injection Paclitaxel 175mg/m2 | 30% |
| Injection Docetaxel 75mg/m2 | 30% |
| Injection Methotrexate 40mg/m2 iv 2 weekly | 20% |
| Oral metronomic | 20% |
| Best supportive care | 50% |

# **Table S5: Common adverse events considered for cost-effectiveness analysis of RT alone and RT combined with Cetuximab**

| Adverse events | Grades | RT alone | RT plus Cetuximab |
| --- | --- | --- | --- |
| Rashes | Grade 1-2 | 9% | 70% |
|  | Grade 3 | 1% | 17% |
|  | Grade 4 | 1% | 17% |
| Hypersensitivity reactions | Grade 3-4 | 2% | 3% |
| Mucositis | Grade 3-4 | 52% | 56% |
| Dysphagia | Grade 3-4 | 30% | 26% |
| Dermatitis | Grade 3-4 | 18% | 23% |
| Xerostomia | Grade 3-4 | 3% | 5% |
| Weight loss | Grade 3-4 | 7% | 11% |
| Pain | Grade 3-4 | 7% | 6% |
| Nausea/Vomitting | Grade 3-4 | 6% | 4% |
| Constipation | Grade 3-4 | 5% | 5% |
| Diarrhoea | Grade 3-4 | 1% | 2% |
| Dyspepsia | Grade 3-4 | 1% | 0% |
| Infection | Grade 3-4 | 1% | 1% |
| Anaemia | Grade 1,2 | 7% | 2% |
| Anaemia | Grade 2-4 | 6% | 1% |

# **Table S6: Common adverse events considered for cost-effectiveness analysis of platinum-based chemotherapy alone and chemotherapy combined with Cetuximab**

| Adverse events | Grades | Chemo plus cetuximab | Chemo alone |
| --- | --- | --- | --- |
| Skin reactions (Rashes) | Grade 3-4 | **9%** | **0.50%** |
| Anorexia (Weight loss) | Grade 3-4 | 6% | 1.50% |
| Nausea/Vomiting | Grade 3-4 | 5% | 3% |
| Febrile Neutropenia | Grade 3-4 | 6% | 7% |
| Neutropenia without fever | Grade 2- 4 | 26% | 31% |
| Anaemia | Grade 3,4 | 14% | 20% |
| Thrombocytopenia | Grade 3-4 | 11% | 12% |
| Leukopenia | Grade 3-4 | 11% | 11% |
| Hypokalaemia | Grade 3-4 | 8% | 5.50% |
| Dyspnoea | Grade 3-4 | 5% | 10% |

# **Table S7: Age-Specific Monthly Probability of Mortality from Natural Causes**

| Age Group (in years) | Monthly Probability of Dying from Natural Causes |
| --- | --- |
| 50–54 | 0.000707 |
| 55–59 | 0.001099 |
| 60–64 | 0.001583 |

# **Figure S1 (a): Loco-regional control curve - RT alone**

# **Figure S1 (b) Loco-regional control curve - RT plus Cetuximab**

# **Figure S2 (a) Progression free survival curve – Chemotherapy alone**

# **Figure S2 (b) Progression free survival – Chemotherapy plus cetuximab**
